# Helicopter emergency medical services missions to islands and the mainland during a 3-year period in Denmark: a population-based study on patient and sociodemographic characteristics, comorbidity, and use of healthcare services

**DOI:** 10.1186/s13049-021-00963-6

**Published:** 2021-10-18

**Authors:** Thea Palsgaard Møller, Annette Kjær Ersbøll, Thora Majlund Kjærulff, Kristine Bihrmann, Karen Alstrup, Lars Knudsen, Troels Martin Hansen, Peter Anthony Berlac, Freddy Lippert, Charlotte Barfod

**Affiliations:** 1 Copenhagen Emergency Medical Services and University of Copenhagen, Copenhagen, Denmark; 2 National Institute of Public Health, University of Southern Denmark, Odense, Denmark; 3 Department of Research and Development, Pre-Hospital Emergency Medical Services, Central Denmark Region, Aarhus, Denmark; 4 Department of Anesthesiology, Aarhus University Hospital, Aarhus, Denmark; 5 The Danish Air Ambulance, Aarhus, Denmark

**Keywords:** Helicopter EMS, Prehospital care, Medical dispatch, Health planning, Resource utilization

## Abstract

**Background:**

The Danish Helicopter Emergency Medical Services (HEMS) is part of the Danish Emergency Medical Services System serving 5.7 million citizens with 1% living on islands not connected to the mainland by road. HEMS is dispatched based on pre-defined criteria including severity and urgency, and moreover to islands for less urgent cases, when rapid transport to further care is needed. The study aim was to characterize patient and sociodemographic factors, comorbidity and use of healthcare services for patients with HEMS missions to islands versus mainland.

**Methods:**

Descriptive study of data from the HEMS database in a three-year period from 1 October 2014 to 30 September 2017. All missions in which a patient was either treated on scene or transported by HEMS were included.

**Results:**

Of 5776 included HEMS missions, 1023 (17.7%) were island missions. In total, 90.2% of island missions resulted in patient transport by HEMS compared with 62.1% of missions to the mainland. Disease severity was serious or life-threatening in 34.7% of missions to islands compared with 65.1% of missions to mainland and less interventions were performed by HEMS on island missions. The disease pattern differed with more “Other diseases” registered on islands compared with the mainland where cardiovascular diseases and trauma were the leading causes of contact. Patients from islands were older than patients from the mainland. Sociodemographic characteristics varied between inhabiting island patients and mainland patients: more island patients lived alone, less were employed, more were retired, and more had low income. In addition, residing island patients had to a higher extend severe comorbidity and more contacts to general practitioners and hospitals compared with the mainland patients.

**Conclusions:**

HEMS missions to islands count for 17.7% of HEMS missions and 90.2% of island missions result in patient transport. The island patients encountered by HEMS are less severely diseased or injured and interventions are less frequently performed. Residing island patients are older than mainland patients and have lower socioeconomic position, more comorbidities and a higher use of health care services. Whether these socio-economic differences result in longer hospital stay or higher mortality is still to be investigated.

**Supplementary Information:**

The online version contains supplementary material available at 10.1186/s13049-021-00963-6.

## Background

Helicopter Emergency Medical Services (HEMS) have been implemented in Denmark to promote better emergency medical care and outcomes for patients living in remote geographical areas. This is to counteract the trend of medical treatment becoming more and more specialized, necessitating centralization of core specialties and specific treatments.

Clinical benefit might be provided by shortening the time to delivery of prehospital care to patients with time critical medical conditions providing necessary specialized medical expertise or equipment to patients before or during transport or providing transport to patients inaccessible by other means of transport [1]. Studies have demonstrated that the use of HEMS is appropriate in terms of an observed high degree of severity of illness or injury for the encountered patients as well as critical interventions being performed in a significant proportion of cases [2, 3]. Even though HEMS has proved beneficial for access to emergency care [4–7], there remains controversy regarding its effect on patient outcomes such as mortality, disability and socioeconomic outcomes [8–10], both overall and within different categories of time-critical diseases [11].

Appropriate use of HEMS seeks to maximize utilization of a scarce resource to improve health outcomes. On islands without a connection to the mainland by road, access to rapid emergency medical care is particularly difficult and here HEMS may essentially be the only possible help. In Denmark, the Danish HEMS system was implemented in 2014 and is now part of the Danish Emergency Medical Services System serving 5.7 million citizens with 1% living on small islands [12]. Knowledge about the population served by the Danish HEMS have been investigated recently in a study presenting baseline characteristics and disease severity of the attended patients [13]. Missions to islands were in this study found to count for 14% of all missions in total. Socioeconomic characteristics of the HEMS population has not yet been described and differences between populations on islands versus mainland are unknown but may add value to health planning and the prioritization of resources.

The aim of this study was to characterize patient and sociodemographic factors, comorbidity and use of healthcare services of patients with HEMS missions to islands versus the mainland.

## Methods

We performed a descriptive study of HEMS missions in a three-year period from 1 October 2014 to 30 September 2017. The results are presented in accordance with the Strengthening the Reporting of Observational Studies in Epidemiology (STROBE) guidelines [14].

### Setting

**Fig. 1:**
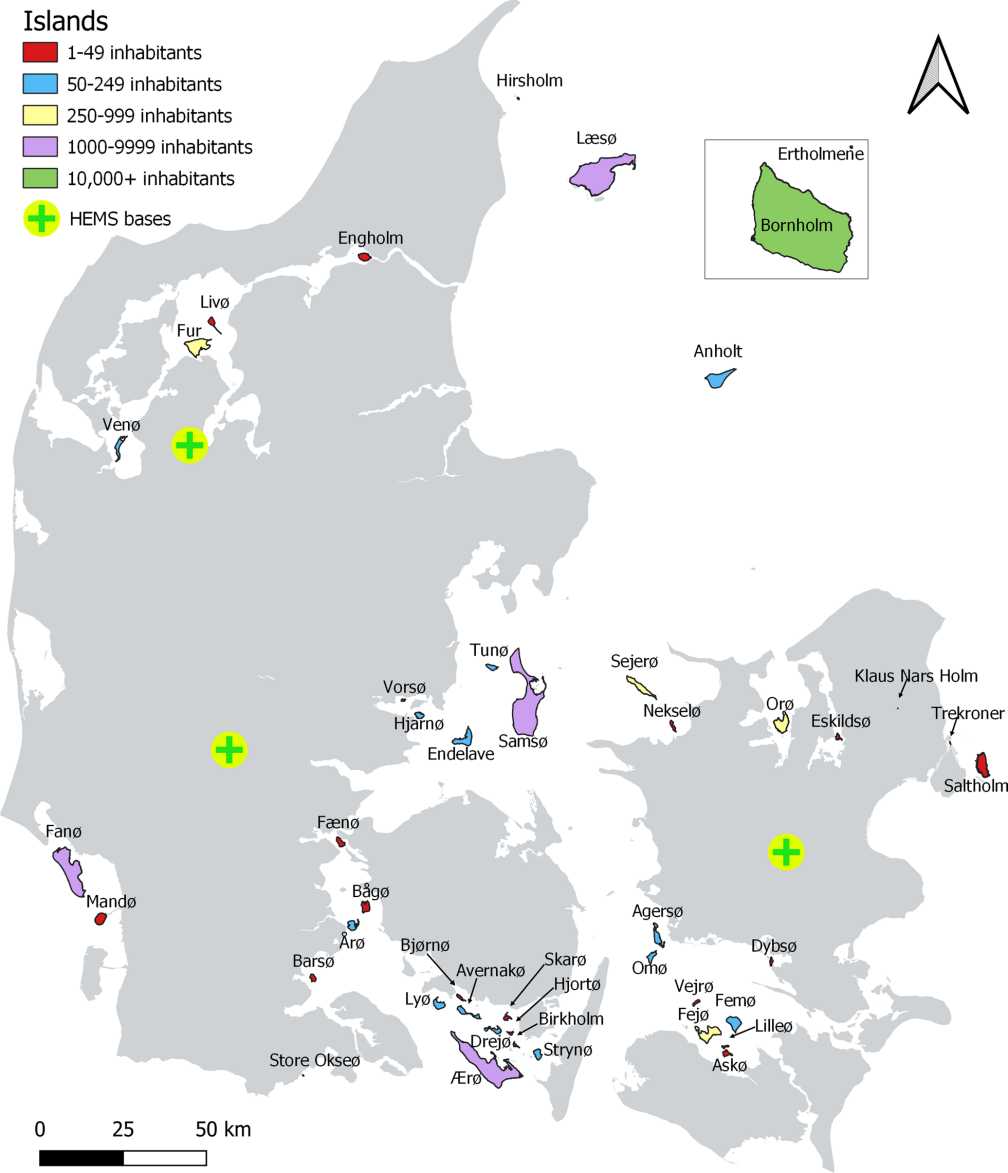
Map of Denmark showing inhabited islands without connection to the mainland by road

Denmark has a population of 5.7 million inhabitants and is a mixed urban, semi-rural and rural country with a total area of approximately 43,000 km^2^. The terrain is mostly flat, and it includes more than 400 islands of which 78 are inhabited. Forty-five of the populated islands are not connected to the mainland by a road and a single populated island, Mandø, is only connected to the mainland by a causeway which is only accessible during low tide [12]. The largest island, Bornholm, is the only island on which there is a hospital with emergency department [15]. A map of Denmark showing inhabited islands without road (on bridge or dam) connection to the mainland is shown in Fig. 1.

In Denmark, medical assistance and admission to hospitals are free of charge. The country is divided into five healthcare regions that have their own emergency medical services (EMS) organization including an Emergency Medical Dispatch Centre (EMDC). Citizens can access emergency care by calling the European emergency phone number 1-1-2. The EMDC dispatch all EMS services and the emergency calls are handled by medical dispatchers (specially trained nurses, emergency medical technicians (EMT) or paramedics). The handling of emergency calls includes a questioning with the caller, assessment of the urgency of the case and activation of the appropriate EMS response. The process is supported by a criteria-based dispatch protocol [16]. Dispatch of HEMS takes place through the EMDC’s based on: (1) emergency calls from citizens (predefined criteria based on type of injury/disease and presumed high urgency of the case), (2) request from pre-hospital healthcare professionals who have already assessed the patient on scene, (3) inquiries from hospitals requesting interhospital patient transports, transport of time-critical donor organs or special medical competence or (4) tasks on islands not connected to the mainland by bridge or dam. For missions to the islands, HEMS can be used for less urgent cases when there is a risk of patient deterioration if alternative transport is the only possibility, with increased risk on mortality or morbidity [17]. Before the implementation of HEMS, patients from islands and the most rural areas were transported by a Search and Rescue Helicopter (without anesthesiology competence) in case of severe disease or injury in remote areas. HEMS covers the entire country 24 h a day 7 days a week by helicopters staffed by a consultant anesthesiologist, a pilot and a specially trained paramedic [13]. There are no other services available for transport from islands to the mainland in case of medical emergencies, such as ambulance boats or similar. In the study period, HEMS operated from three different bases (Ringsted, Billund, Skive, please refer to Fig. 1).

### Patient selection

All missions in which a patient was either treated on scene or transported by HEMS in the study period were included. Invalid registrations (e.g., registered test cases) were excluded. Cases in which more than one patient was seen or treated was excluded (n = 308) because patient related data in these cases were of poor quality, with respect to the purpose of the study. Telephone contacts, aborted missions (missions which were cancelled after take-off) and rejected missions (missions that were not dispatched due to specific reasons such as weather under HEMS minima or technical reasons) were excluded. Furthermore, interhospital transports were excluded.

### Data sources and data processing

Data on HEMS missions were obtained from the HEMS organization, who administer the Danish HEMS database containing information on nearly all missions dispatched by the five regional EMDCs in Denmark. The database includes geographical and operational data, patient specific data and treatment related data. A thorough description of the database is presented in a recent paper [18].

Residential locations at inhabited islands with no connection to the mainland by road were identified from the Danish Address Register [19]. Islands were categorized into five categories by population size 1 January 2015 (1–49, 50–249, 250–999, 1000–9999 and ≥ 10,000 inhabitants) [12]. Data on residential locations were transferred to Statistics Denmark and linked to the remaining data by a unique address identification number.

Data from the HEMS missions were linked with data from the Danish nationwide population registers at Statistics Denmark by use of the unique personal identification number assigned to each resident of Denmark at birth or immigration [20]. Information on demography, country of origin, cohabitation status, and residential location was obtained from the Danish Civil Registration System [20]. Data on socioeconomic position was available from Danish registers on personal labor market affiliation [21], education [22] and income [23]. Information on comorbidity and hospital contacts were obtained from the Danish National Patient Register [24] and contact to the general practitioner was obtained from the Danish National Health Service Register [25].

### Derived variables and definitions

HEMS missions were divided into mainland and island missions based on information on the geographical destination listed in the HEMS database [18]. In addition, information on whether the transported patient was living on the island 1 January of the same year as the HEMS mission was obtained from Statistics Denmark based on the previous described data sources. HEMS Missions were furthermore categorized into two categories: “attended” missions in which the patient received treatment from the HEMS staff and was either completed at scene or transported to hospital by ground EMS, and “transported” missions in which the patient was transported by HEMS. Time and season variables were derived from the time of the HEMS alarm registered automatically in the database [18]. Time of day were divided according to working hours: daytime (7 a.m.–3 p.m.), evening (4 p.m.–11 p.m.), and nighttime (12 a.m.–6 a.m.).

**Fig. 2:**
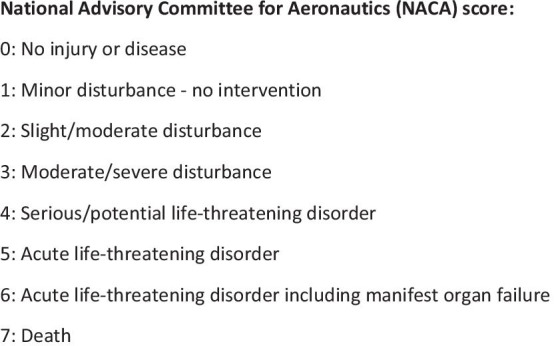
Box explaining the NACA score

Clinical variables were obtained from the HEMS database and described in the following. The severity of the disease was reflected by the registered National Advisory Committee for Aeronautics (NACA) score [26] which is registered by the HEMS staff and defined as shown in Fig. 2. We considered a NACA score of 4–7 to represent a patient with need for hospitalization and in a critical condition corresponding to a severe or critical illness or injury. Based on this consideration, we further dichotomized the NACA score into “Critical condition” (NACA score 4–7) and “non-critical condition” (NACA score 0–3), as done in a recent study [13]. Disease category, as registered by the HEMS team, was extracted directly from the dataset. This variable was reduced to four categories: “cardiovascular disease”, “trauma”, “neurology” and “other diseases.” The latter category was a merge of original disease categories that had very few cases (such as “unspecified medical diseases”, gastrointestinal diseases, respiratory diseases or burns). Critical interventions performed by the HEMS team were intubation, blood transfusion, intraosseous access, mechanical chest compression. Ultrasound examination were also included as a critical investigation of e.g. intraabdominal fluid, pneumothorax or cardiac tamponade, affecting the triage of patients to specific hospitals with relevant competencies.

Age group (0–15, 16–30, 31–65 and ≥ 66 years), sex, cohabitation status (cohabiting versus living alone) were derived for 1 January at the year of the HEMS mission. Patient comorbidity was based on Charlson Comorbidity Index score [27] at three levels (0: no comorbidity; 1: mild comorbidity; and ≥ 2: severe comorbidity) based on hospital contacts within a 10-year period before the year of the HEMS mission. Contacts to the hospital or general practitioner was measured as at least one contact within the year before the HEMS mission. Socioeconomic position was measured for the adult population (≥ 18 years). Educational level was categorised as elementary school (< 10 years of education); short education (10–12 years of education), or medium/long education (> 12 years of education) and the information was obtained on 1 January at the year of the HEMS mission. To make income comparable and capture family size and income fluctuations over the lifespan, we divided the equalised disposable household income into income quintiles and stratified in three age groups (18–30, 31–65, > 65 years). A similar income measure was used in another Danish study [28]. Employment was defined according to the individual’s work potential and grouped in five levels: “employed” (including employed or receiving unemployment insurance); “unemployed” (including unemployed for more than six months, or receiving social security or early retirement); “students”; and “retired” (receiving state pension or being voluntarily early retired) and “other”. Few individuals had no information on employment status (3.1%) and these were included in the “retired” category if they were aged 65 years or above or in the “unemployed” category if they were younger than 65 years. Income and employment status were obtained from the year before the HEMS mission.

### Statistical analyses

Descriptive analyses were performed by use of numbers and percentages. HEMS missions were described by type of mission, time, season, severity, disease category and clinical interventions performed. The HEMS missions that were linkable with data from the nationwide population registers at Statistics Denmark were described by patient and sociodemographic characteristics, comorbidity and use of healthcare services. HEMS patients were described overall and across missions to islands versus missions to the mainland. As a supplementary analysis, the sociodemographic profile of the general island and mainland population residing Denmark 1 January 2016 was tabulated to enable a comparison with the general Danish population. Data management and statistical analyses were performed by use of Excel and SAS version 9.4. The map (Fig. 1) was performed in Q-GIS version 3.

## Results

**Fig. 3:**
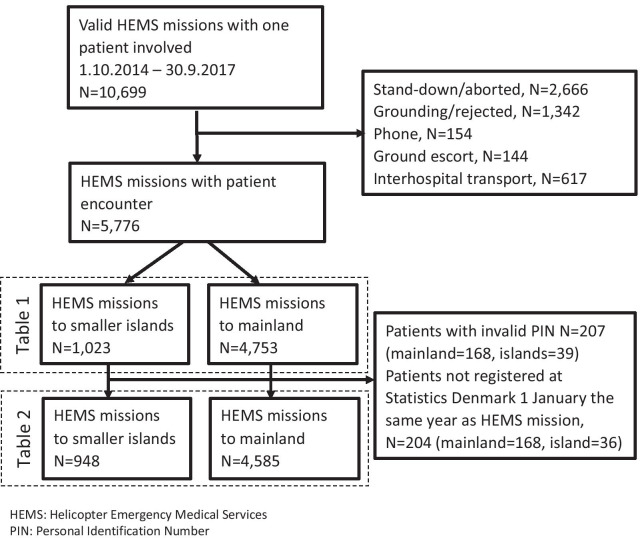
Study flowchart

Of 10,699 valid HEMS missions with a single patient involved in the study period, a total of 5776 (54.0%) HEMS missions were completed with patient encounter and were included in the study (Fig. 3). Of those 1023 (17.7%) were missions to islands.

### HEMS mission characteristics

Characteristics of HEMS missions to islands and the mainland are illustrated in Table 1. The proportion of transported patients was 90.2% for island missions compared with 62.1% for mainland missions. The median duration (Q1,Q3) of missions to islands were 113 min [Q1,Q3: 94,138] whereas the medium duration (Q1,Q3) of missions to the mainland were 98 min [Q1,Q3: 61,128].

**Table 1:** Characteristics of HEMS missions to islands and the mainland in the period 1 October 2014–30 September 2017, N (%)

Variable	Islands N = 1,023	Mainland N = 4,753	Total N = 5,776
Missions, total			
Attended Transported	100 (9.8) 923 (90.2)	1,802 (37.9) 2,951 (62.1)	1,902 (32.9) 3,874 (67.1)
Mission duration in minutes, median (Q1,Q3)	113 [94,138]	98 [61,128]	–
Season			
Autumn Winter Spring Summer	228 (22.3) 138 (13.5) 255 (24.9) 402 (39.3)	1,080 (22.7) 851 (17.9) 1,356 (28.5) 1,466 (30.8)	1,308 (22.7) 989 (17.1) 1,611 (27.9) 1,868 (32.3)
Time of day (shift)			
Daytime Evening Nighttime	494 (48.3) 394 (38.5) 135 (13.2)	2,665 (56.1) 1,617 (34.0) 471 (9.9)	3,159 (54.7) 2,011 (34.8) 606 (10.5)
NACA score as assessed by HEMS staff			
0 No injury or disease, missing 1 Minor disturbance—no intervention 2 Slight/moderate disturbance 3 Moderate/severe disturbance 4 Serious/potential life-threatening disorder 5 Acute life-threatening disorder 6 Acute life-threatening disorder incl manifest organ failure 7 Death	5 (0.5) 20 (2.0) 120 (11.7) 523 (51.1) 264 (25.8) 42 (4.1) 26 (2.5) 23 (2.2)	15 (0.3) 70 (1.5) 333 (7.0) 1,240 (26.1) 1,609 (33.9) 543 (11.4) 474 (10.0) 469 (9.9)	20 (0.4) 90 (1.6) 453 (7.8) 1,763 (30.5) 1,873 (32.4) 585 (10.1) 500 (8.7) 492 (8.5)
Naca score as assessed by HEMS staff, dichotomized			
NACA 0–3, non-critical emergency, total NACA 0–3, non-critical emergency, island tourists NACA 0–3, non-critical emergency, island inhabitants NACA 4–7, critical emergency, total NACA 4–7, critical emergency, island tourists NACA 4–7, critical emergency, island inhabitants	668 (65.3) 199 (71.6) 426 (63.3) 355 (34.7) 79 (28.4) 244 (36.4)	1658 (34.9) – – 3095 (65.1) – –	2326 (40.3) – – 3450 (59.7) – –
Disease category			
Cardiovascular Trauma Neurology Other Missing	285 (27.9) 157 (15.4) 154 (15.1) 356 (34.8) 71 (6.9)	2,030 (42.7) 1,212 (25.5) 766 (16.1) 591 (12.4) 154 (3.2)	2,315 (40.1) 1,369 (23.7) 920 (15.9) 947 (16.4) 225 (3.9)
Interventions performed during contact*			
Intubation Blood transfusion I.O access Ultrasound examination Mechanical chest compression Thoracotomy (too few to report)	61 (6.0) 7 (0.7) 10 (1.0) 82 (8.0) 21 (2.1)	1,051 (22.1) 141 (3.0) 321 (6.8) 1,125 (23.7) 379 (8.0)	1,112 (19.3) 148 (2.6) 331 (5.7) 1,207 (20.9) 400 (6.9)

*Percentages of cases that had the intervention performed (does not add up to 100%)

HEMS missions were overall more frequent during summer and daytime with slightly more island missions during summer and fewer during daytime compared with mainland missions. Disease severity, measured with NACA, was lower for island patients compared with mainland patients (34.7% versus 65.1% had critical emergency measured by NACA 4–7). Inhabiting island patients had higher NACA score compared to “tourist patients.”

The disease pattern also differed. Thus, the most frequent disease categories were “other” (34.8%) and cardiovascular disease (27.9%) among island patients whereas cardiovascular disease and trauma counted for respective 42.7% and 25.5% among mainland patients. Approximately the same proportion of patients from islands and the mainland had neurological diseases. Overall, clinical interventions, performed by HEMS staff, were less frequently used for island patients compared with mainland patients.

### Sociodemographic factors, comorbidity and use of healthcare services

The number of island patients who were permanent residents on the island was 670 (70.7%) out of 948 total island patients (Table 2). When comparing the residing island patients with the mainland patients, differences in both patient and sociodemographic characteristics, comorbidity and use of healthcare services were demonstrated.

**Table 2:** Patient characteristics in HEMS missions to islands and the mainland in the period 1 October 2014–30 September 2017, N (%)

Variable	Islands			Mainland		Total N = 5,365	
	Missions N = 948		Background population January 2016 N = 59,361	Missions N = 4,417	Background population January 2016 N = 5,647,891	Missions N = 5,365	Background population January 2016 (total) N = 5,707,252
	“Tourists” N = 278	Inhabitants N = 670					
Gender							
Female Male	109 (39.2) 169 (60.8)	274 (40.9) 396 (59.1)	29,819 (50.2) 29,542 (49.8)	1,426 (32.3) 2991 (67.7)	2,839,545 (50,3) 2,808,346 (49,7)	1,809 (33.7) 3,556 (66.3)	2,869,364 (50.3) 2,837,888 (49.7)
Agegroup							
0–15 16–30 31–65 66 +	27 (9.7) 32 (11.5) 124 (44.6) 95 (34.2)	27 (4.0) 24 (3.6) 242 (36.1) 377 (56.3)	8,277 (13.9) 6,951 (11.7) 27,610 (46.5) 16,523 (27.8)	359 (8.2) 457 (10.4) 1,979 (44.8) 1,622 (36.7)	1,021,124 (18,1) 1,090,562 (19,3) 2,544,698 (45,1) 991,507 (17,6)	413 (7.7) 513 (9.6) 2,345 (43.7) 1094 (39.0)	1,029,401 (18.0) 1,097,513 (19.2) 2,572,308 (45.1) 1,008,030 (17.7)
Cohabitation status							
Cohabiting Living alone	192 (69.1) 86 (30.9)	395 (59.0) 275 (41.0)	38,569 (65.0) 20,792 (35,0)	2,829 (64.0) 1,588 (36.0)	3,759,970 (66.6) 1,887,921 (33,4)	3,416 (63.7) 1,949 (36.3)	3,798,539 (66.6) 1,908,713 (33.4)
Employment*							
Employed Unemployed Student Retired Other	102 (42.5) 30 (12.5) ** 99 (41.3) 9 (3.8)	127 (20.0) 111 (17.5) ** 384 (60.4) 14 (2.2)	22,657 (45,6) 6,855 (13,8) 1,460 (2,9) 17,664 (35,5) 1,063 (2,1)	1,520 (38.0) 652 (16.3) ** 1,667 (41.7) 156 (3.9)	2,417,643 (53.8) 583,963 (13.0) 328,923 (7.3) 1,027,813 (22.9) 131,751 (2.9)	1,749 (35.9) 793 (16.3) ** 2,150 (44.1) 179 (3.7)	2,440,300 (53.8) 590,818 (13.0) 330,383 (7.3) 1,045,477 (23.0) 132,814 (2.9)
Education*							
Elementary Short Medium/long Missing	57 (23.8) 102 (42.5) 75 (31.3) 6 (2.5)	276 (43.4) 245 (38.5) 83 (13.1) 32 (5.0)	18,147 (36,5) 22,671 (45,6) 8.881 (17,9) -	1,663 (41.6) 1,707 (42.7) 480 (12.0) 145 (3.6)	1,400,470 (31.2) 2,014,787 (44,9) 1,074,836 (23,9) -	1,996 (41.0) 2,054 (42.2) 638 (13.1) 183 (3.8)	1,418,617 (31.2) 2,037,458 (44.9) 1,083,717 (23.9) -
Income quintiles*							
1, (lowest) 2 3 4 5 (highest)	39 (16.3) 53 (22.1) 37 (15.4) 53 (22.1) 58 (24.2)	210 (33.0) 197 (31.0) 102 (16.0) 83 (13.1) 44 (6.9)	12,544 (25,2) 12,124 (24,4) 10,413 (21,0) 8,744 (17,6) 5,874 (11,8)	1099 (27.5) 992 (24.8) 753 (18.8) 654 (16.4) 497 (12.4)	895,419 (19.9) 895,831 (20.0) 897,546 (20.0) 899,214 (20.0) 902,083 (20.1)	1,348 (27.7) 1,242 (25.5) 892 (18.3) 790 (16.2) 599 (12.3)	907,963 (20.0) 907,955 (19.8) 907,959 (20.0) 907,958 (20.0) 907,957 (20.0)
Comorbidity							
None Mild Severe	180 (64.7) 50 (18.0) 48 (17.3)	310 (46.3) 130 (19.4) 230 (34.3)	47,243 (79,6) 6,114 (10,3) 6,004 (10,1)	3,010 (68.1) 607 (13.7) 800 (18.1)	4,806,843 (85.1) 446,932 (7.9) 394,116 (7.0)	3,500 (65.2) 787 (14.7) 1078 (20.1)	4,854,086 (85.1) 453,046 (7.9) 400,120 (7.0)
GP contact the year before HEMS mission							
Yes No	248 (89.2) 30 (10.8)	619 (92.4) 51 (7.6)	49,417 (83,2) 9,944 (16,8)	3,872 (87.7) 545 (12.3)	4,654,682 (82.4) 993,209 (17.6)	4,739 (88.3) 626 (11.7)	4,704,099 (82.4) 1,003,153 (17.6)
Hospital contact the year before HEMS mission							
Yes No	163 (58.6) 115 (41.4)	437 (65.2) 233 (34.8)	30,093 (50,7) 29,268 (49,3)	2,494 (56.5) 1,923 (43.5)	2,373,605 (42.0) 3,274,286 (58.0)	3,094 (57.7) 2,271 (42.3)	2,403,698 (42.1) 3,303,554 (57.9)

Employment, education and income are tabulated for the adult population (≥ 18 years, N = 4,871 (HEMS missions)/ N = 4,539,792 (background population))

**Students were included in the “other category”, as there were too few cases to report

Island patients were older compared with mainland patients and 59.1% among island patients compared with 67.7% among mainland patients were males. The cohabitation status was different for island and mainland patients (41.0% versus 36.0% were living alone, respectively). Employment also differed so that 20.0% were employed among island patients in contrast to 38.0% among mainland patients. In addition, due to the different age distributions a higher proportion of the island patients were retired (60.4% versus 41.7%). The educational level was almost similar among HEMS patients, but the income level differed with only 6.9% of patients with the highest income quintile among island patients, compared with 12.4% of mainland patients.

In terms of comorbidity, the proportion of patients with severe comorbidity according to Charlson Comorbidity Index score differed (34.3% versus 18.1% of island and mainland patients, respectively). The use of healthcare services was however only slightly higher for island patients, who had at least one general practitioner (GP) or hospital contact within the year before the HEMS mission in 92.4% and 65.2% of cases compared with 87.7% and 56.5% among mainland missions, respectively.

As also seen in Table 2, there seems to be a relatively larger difference between patients and background population on islands compared to the mainland populations for some variables. This is true for age, where island patients are relatively older than the background population compared to the mainland population, employment with relatively more retired patients on islands, comorbidity with a relatively higher number of patients with severe comorbidity, and visits to the GP.

When comparing the overall island population (including both residing island patients and the “tourists” that might affect the results) with the mainland population, the sociodemographic differences were less evident. In this case, the cohabiting status were more equal, more island patients had medium or long education than the mainland patients and the income level was almost the same for island patients and mainland patients. In addition, the differences in comorbidity and use of healthcare services were less pronounced.

Sociodemographic characteristic of the general population of Denmark 1 January 2016 living on islands versus the mainland is presented in Additional file 1: Appendix.

## Discussion

This study aimed to characterize HEMS missions and the patient population for missions to islands and the mainland focusing on differences in patient and sociodemographic characteristics, comorbidity and use of healthcare services. The island patient population was older, had lower disease severity, another disease pattern in the acute contact, and fewer interventions performed by HEMS compared with mainland patients. Despite this, the proportion of patients transported by HEMS from islands was higher compared with patients from the mainland. The socioeconomic position was lower among residing island patients and they had more severe comorbidities and more GP and hospital contacts compared with the mainland patients.

### Disease severity and HEMS interventions

We included 54% of all HEMS enquiries in the study period. This proportion of missions with patient encounter as well as our findings of temporal trends for the missions are comparable with other reported results [3, 29]. The finding of a lower acute disease severity for the island patients was expected given the dispatch criteria that HEMS should be dispatched to islands in less urgent cases when there is a risk of patient deterioration if alternative transport is the only possibility, with increased risk on mortality or morbidity. Consequently, lesser interventions were being performed in these missions. No other studies have addressed the island population even though many countries have the same geographical composition. Based on the results and in light of the fact that the time-saving factor may in many cases be the most important compared to the small gain in intervention opportunities, one consideration could be whether the staff composition is always correct or whether differentiated missions could be an option, for example based on the dispatch criterion provided by the dispatch center. Another fact to consider is that while we send HEMS to low acuity cases on islands (and with longer mission durations), other patients on the mainland with life threatening conditions may lack the opportunity for fast emergency care provided by HEMS. The extent of this problem could be explored in future studies.

### Socioeconomic variables

We found lower socioeconomic status among residing island patients compared with mainland patients and compared to the background populations, patients’ age and employment seemed to be more unequally distributed on islands. This result is interesting in terms of healthcare planning not only on islands but in remote areas in general. It is well known that differences in socioeconomic characteristics such as education, income and employment exist across geographical areas [12]. In general, the socioeconomic position is higher among the population living in the surrounding of the larger cities. Therefore, the results were somehow expected and corresponds with the lower socioeconomic position observed among the general population living on islands compared with the mainland in Denmark (see also Additional file 1: Appendix/supplemental table). Reasons are unknown since no other studies have explored socioeconomic characteristics on island populations.

Interestingly, when looking at the entire population of island patients including both residing patients and tourists (comprising one third of the island patients), the socioeconomic differences seemed to level out, demonstrating that we are dealing with two completely different types of populations. The socioeconomic differences found in the island patient population may be typical for the urban–rural gradient. This underlines a complexity in health planning and the question of appropriateness of the care offered. In general, the total HEMS population had lower socioeconomic position than the average population of Denmark.

### Comorbidity and contact to healthcare services

Poor physical health is related to age, employment, education and marital status [30]. Therefore, it is not surprising that the island patients have a higher degree of comorbidity than on the mainland, since the proportion of the elderly is higher. From a geographical point of view, this is also expected since geographical differences in disease occurrence exist across Denmark [31, 32]. However, no previous geographical epidemiological studies have had focus on comparing the socioeconomic position and health status across island and mainland populations. We found a more frequent use of GP and hospitals services for the residing island patients compared with the mainland patients. However, given the large difference in age and comorbidity, it could be argued that the use of healthcare services is relatively lower than expected. This may be explained by the more difficult access to hospitals and GPs on islands, implying that people see a doctor less. Also, health behavior may be different among island patients. However, the use of GP and hospital services measured in the present study was not adjusted for healthcare needs or health behavior, so reasons can only be hypothesized and could be explored further.

### Implications of this study and future perspectives

This is the first study to address an island population encountered by HEMS in comparison with missions to the mainland. In optimizing and targeting treatment, it is important to know the characteristics of the served population. HEMS missions to the islands make up a large proportion of all HEMS missions in Denmark. We found that 90% of the island missions resulted in a patient transport, demonstrating the appropriateness of the HEMS dispatch despite lower disease severity. Given the lack of other transport options for these people, dispatch of HEMS may be lifesaving even with less severity of disease or injury or simply the only transportation possible in reasonable time. Denmark is a small country and the distances are small, but still we have a complex infrastructure with ferry crossings with a long time between departures. The islands are thus separated in time from the larger hospitals, which leads to an inequality in access to emergency health care, which needs to be considered politically. Further research could elaborate and refine specific diagnoses and circumstances necessitating transport by HEMS in contrast to transport in own vehicle by ferry.

Even if the condition on islands are considered less serious and less interventions are performed, it is possible that the mortality of the group residing on islands is higher due to the differences in socio-economic factors and comorbidity. This must be investigated in more detail by exploring the patients’ length at stay at hospital and mortality after admission from an island. In addition, other geographical aspects could be explored, not only looking at islands, but also other rural areas with longer distances to healthcare facilities like done in a recent study that found lack of rapid transport opportunities in terms of HEMS as the primary cause of lost life years in a specific geographical area [33]. In contrast, another study found no survival benefit for trauma patients transported by HEMS versus ground EMS, even when geographical factors such as distance were taken into account [34].

### Strengths and limitations

A major strength of this study is the nationwide population-based study design including all three HEMS units providing a complete picture of the Danish HEMS patient population. The data quality is considered high as reported in a recent study [18]. In Denmark, we also have the unique opportunity to access information on socioeconomic measures based on individual-level data, allowing comprehensive register studies. We were able to use data linkage due to the PIN number registered in a large proportion of the HEMS missions. However, the study also has limitations. The investigated population represents HEMS missions with patient encounter. However, the characteristics of patients that did not have direct contact with HEMS might differ from the investigated population, possibly affecting the interpretation of the results for the island patients in comparison with the mainland patients. Finally, the study was a nationwide study performed in Denmark and the results reflect a Danish pattern regarding the citizens utilization of the healthcare system, which may depend on traditions and health behaviour. It also represents the Danish prehospital setting. This might decrease the generalizability of the identified patterns in an international perspective. However, the results may be useful in countries with comparable demography, geography, island populations and infrastructure.

## Conclusion

HEMS missions to islands count for 17.7% of HEMS missions and 90.2% of island missions result in patient transport. The island patients encountered by HEMS are less severely diseased or injured and interventions are less frequently performed compared with the mainland patients. Residing island patients are older than mainland patients and have lower socioeconomic position, more comorbidities and a higher use of health care services. Whether these socio-economic differences result in longer hospital stay or higher mortality is still to be investigated.

## Supplementary Information

**Additional file 1: Table**. Sociodemographic characteristic of the general population of Denmark 1 January 2016 living on islands (categorised into 1–49, 50–249, 250–999, 1000–9999 and ≥10,000 inhabitants) versus mainland (including island connected to the mainland by road), N (%)

## Data Availability

Not applicable.

## Abbreviations

HEMS: Helicopter Emergency Medical Services
NACA: National Advisory Committee for Aeronautics
STROBE: Strengthening the Reporting of Observational Studies in Epidemiology
EMS: Emergency Medical Services
EMT: Emergency Medical Technicians
EMDC: Emergency Medical Dispatch Centre
GP: General Practitioner
