# Supplementary material for: Helicopter emergency medical services missions to islands and the mainland during a 3-year period in Denmark: a population-based study on patient and sociodemographic characteristics, comorbidity, and use of healthcare services
Source: Scand J Trauma Resusc Emerg Med. 2021 Oct 18;29:152. doi: 10.1186/s13049-021-00963-6 (PMC8522108; doi:10.1186/s13049-021-00963-6)
Supplement: Supplementary file 1 — Additional file 1: Table. Sociodemographic characteristic of the general population of Denmark 1 January 2016 living on islands (categorised into 1–49, 50–249, 250–999, 1000–9999 and ≥10,000 inhabitants) versus mainland (including island connected to the mainland by road), N (%) [file 13049_2021_963_MOESM1_ESM.docx]

| **Appendix**. Sociodemographic characteristic of the general population of Denmark 1 January 2016 living on islands (categorised into 1–49, 50–249, 250–999, 1,000–9,999 and ≥10,000 inhabitants)) versus mainland (including island connected to the mainland by road), N (%) | | | | | | | |
| --- | --- | --- | --- | --- | --- | --- | --- |
| **Variable** | Categories | Islands | | | | | Mainland (including islands connected to mainland by road) |
|  |  | 1–49 | 50–249 | 250–999 | 1,000–9,999 | ≥10,000 inhabitants (Bornholm) |  |
| **Gender** | Female | 136 (43.6) | 901 (48.0) | 1,187 (49.3) | 7,541 (50.0) | 20,054 (50.5) | 2,839,545 (50.3) |
|  | Male | 176 (56.4) | 978 (52.0) | 1,221 (50.7) | 7,529 (50.0) | 19,638 (49.5) | 2,808,346 (49.7) |
| **Age group** | 0-15 | 23 (7.4) | 196 (10.4) | 263 (10.9) | 1,964 (13.0) | 5,831 (14.7) | 1,021,124 (18.1) |
|  | 16-30 | 33 (10.6) | 125 (6.7) | 189 (7.8) | 1,534 (10.2) | 5,070 (12.8) | 1,090,562 (19.3) |
|  | 31-65 | 156 (50.0) | 861 (45.8) | 1,100 (45.7) | 6,867 (45.6) | 18,626 (46.9) | 2,544,698 (45.1) |
|  | 66+ | 100 (32.1) | 697 (37.1) | 856 (35.5) | 4,705 (31.2) | 10,165 (25.6) | 991,507 (17.6) |
| **Cohabitation status** | Cohabiting | 197 (63.1) | 1,200 (63.9) | 1,479 (61.4) | 9,744 (64.7) | 25,949 (65.4) | 3,759,970 (66.6) |
|  | Living alone | 115 (36.9) | 679 (36.1) | 929 (38.6) | 5,326 (35.3) | 13,743 (34.6) | 1,887,921 (33.4) |
| **Employment*** | Employed | 126 (45.5) | 684 (41.3) | 858 (40.8) | 5,733 (45.0) | 15,256 (46.4) | 2,417,643 (53.8) |
|  | Unemployed | 25 (9.0) | 174 (10.5) | 246 (11.7) | 1,492 (11.7) | 4,918 (14.9) | 583,963 (13.0) |
|  | Student | 12 (4.3) | 26 (1.6) | 34 (1.6) | 359 (2.8) | 1,029 (3.1) | 328,923 (7.3) |
|  | Retired | 102 (36.8) | 715 (43.2) | 901 (42.9) | 4,890 (38.3) | 11,056 (33.6) | 1,027,813 (22.9) |
|  | Other | 12 (4.3) | 58 (3.5) | 62 (3.0) | 278 (2.2) | 653 (2.0) | 131,751 (2.9) |
| **Education*** | Elementary ** | 106 (38.3) | 558 (33.7) | 823 (39.2) | 4,390 (34.4) | 12,270 (37.3) | 1,400,470 (31.2) |
|  | Short | 115 (41.5) | 674 (40.7) | 897 (42.7) | 5,787 (45.4) | 15,198 (46.2) | 2,014,787 (44.9) |
|  | Medium/long | 56 (20.2) | 425 (25.6) | 381 (18.1) | 2,575 (20.2) | 5,444 (16.5) | 1,074,836 (23.9) |
| **Income quintiles*** | 1 (lowest) | 98 (35.4) | 456 (27.5) | 591 (28.1) | 3,050 (23.9) | 8,349 (25.4) | 895,419 (19.9) |
|  | 2 | 46 (16.6) | 372 (22.5) | 490 (23.3) | 2,855 (22.4) | 8,361 (25.4) | 895,831 (20.0) |
|  | 3 | 46 (16.6) | 315 (19.0) | 424 (20.2) | 2,523 (19.8) | 7,105 (21.6) | 897,546 (20.0) |
|  | 4 | 46 (16.6) | 281 (17.0) | 347 (16.5) | 2,400 (18.8) | 5,670 (17.2) | 899,214 (20.0) |
|  | 5 (highest) | 41 (14.8) | 233 (14.1) | 249 (11.9) | 1,924 (15.1) | 3,427 (10.4) | 902,083 (20.1) |
| **Comorbidity** | None | 238 (76.3) | 1,420 (75.6) | 1,816 (75.4) | 12,072 (80.1) | 31,697 (79.9) | 4,806,843 (85.1) |
|  | Mild | 39 (12.5) | 221 (11.8) | 271 (11.3) | 1,479 (9.8) | 4,104 (10.3) | 446,932 (7.9) |
|  | Severe | 35 (11.2) | 238 (12.7) | 321 (13.3) | 1,519 (10.1) | 3,891 (9.8) | 394,116 (7.0) |
| **GP contact the year before 1 January 2016** | Yes | 265 (84.9) | 1,599 (85.1) | 2,064 (85.7) | 12,375 (82.1) | 33,114 (83.4) | 4,654,682 (82.4) |
|  | No | 47 (15.1) | 280 (14.9) | 344 (14.3) | 2,695 (17.9) | 6,578 (16.6) | 993,209 (17.6) |
| **Hospital contact the year before 1 January 2016** | Yes | 141 (45.2) | 878 (46.7) | 1,060 (44.0) | 6,975 (46.3) | 21,039 (53.0) | 2,373,605 (42.0) |
|  | No | 171 (54.8) | 1,001 (53.3) | 1,348 (56.0) | 8,095 (53.7) | 18,653 (47.0) | 3,274,286 (58.0) |
| ***** Employment, education and income are tabulated for the adult population (≥18 years); ** Missing values were included in the “elementary”, as there were too few cases to report; GP: General Practitioner | | | | | | | |
